# Unexpected Outcomes of Renal Function after Radical Nephrectomy: Histology Relevance along with Clinical Aspects

**DOI:** 10.3390/jcm10153322

**Published:** 2021-07-28

**Authors:** Federico Di Marco, Antonello Pani, Matteo Floris, Alberto Martini, Giacomo Dell’Antonio, Umberto Capitanio, Arianna Bettiga, Alessandro Larcher, Alessandra Cinque, Roberto Bertini, Alberto Briganti, Andrea Salonia, Francesco Montorsi, Francesco Trevisani

**Affiliations:** 1 Urological Research Institute (URI), Division of Experimental Oncology, IRCCS San Raffaele Scientific Institute, 20132 Milan, Italy; dimarco.federico@hsr.it (F.D.M.); capitanio.umberto@hsr.it (U.C.); bettiga.arianna@hsr.it (A.B.); larcher.alessandro@hsr.it (A.L.); cinque.alessandra@hsr.it (A.C.); bertini.roberto@hsr.it (R.B.); briganti.alberto@hsr.it (A.B.); salonia.andrea@hsr.it (A.S.); montorsi.francesco@hsr.it (F.M.); 2 Nephrology, Dialysis and Transplantation, Università Degli Studi di Cagliari, G. Brotzu Hospital, 09134 Cagliari, Italy; antonellopani@aob.it (A.P.); matteo.floris@aob.it (M.F.); 3 Unit of Urology, IRCCS San Raffaele Scientific Institute, 20132 Milan, Italy; martini.alberto@hsr.it; 4 Unit of Pathology, IRCCS San Raffaele Scientific Institute, 20132 Milan, Italy; dellantonio.giacomo@hsr.it

**Keywords:** AKI, CKD, eGFR, radical nephrectomy, renal pathology

## Abstract

Acute kidney injury (AKI) and chronic kidney disease (CKD) are common events after radical nephrectomy (RN). In this study we aimed to predict AKI and CKD after RN relying on specific histological aspects. We collected data from a cohort of 144 patients who underwent radical nephrectomy. A histopathological review of the healthy part of the removed kidney was performed using an established chronicity score (CS). Logistic regression analyses were performed to predict AKI after RN, while linear regression analysis was adopted for estimated glomerular filtration rate (eGFR) variation at 1 year. The outcomes of the study were to determine variables correlated with AKI onset, and with eGFR decay at 1 year. The proportion of AKI was 64%. Logistic analyses showed that baseline eGFR independently predicted AKI (odds ratio 1.04, 95%CI 1.02:1.06). Moreover, AKI (Beta −16, 95%CI −21:−11), baseline eGFR (Beta −0.42, 95%CI −0.52:−0.33), and the presence of arterial narrowing (Beta 10, 95%CI 4:15) were independently associated with eGFR decline. Our findings showed that AKI onset and eGFR decline were more likely to occur with higher baseline eGFR and lower CS, highlighting that RN in normal renal function patients represents a more traumatic event than its CKD counterpart.

## 1. Introduction

Acute kidney injury (AKI) and chronic kidney disease (CKD) are two possible sequelae following radical nephrectomy (RN) [1]. Post-surgical rates of morbidity and mortality related to kidney imbalance have been thoroughly investigated both from urological and nephrological perspectives [2]. The former focused on how surgery (radical vs. partial nephrectomy) and technique (cold ischemia vs. warm ischemia vs. no ischemia) affect renal function, cardiovascular performance, and survival outcomes [3,4]. The latter, instead, investigated the glomerular filtration rate decline after RN comparing the oncological patients affected by kidney cancer with the living kidney donors (LKDs), elucidating the differences in terms of AKI incidence and CKD development over time [5,6]. 

Although numerous efforts have been made by clinicians to better understand and predict the development of AKI and CKD after RN, several problems remain unsolved. One of the most intriguing arguments is that AKI and CKD occurring after surgery should be considered differently in respect to other clinical settings when renal function decline is sharply compromised but not related to an acute loss of nephron mass [7]. At the same time, controversy exists over whether to apply the current CKD classification—which historically applies to nephrological patients with two kidneys—to patients who undergo radical nephrectomy [8]. Finally, the importance of the histological characteristics of the removed kidney parenchyma in predicting functional outcomes is still unclear.

Thus, to address these points, we carried out an in-depth analysis on the clinical and pathological characteristics of 144 consecutive RN cases in predicting AKI and CKD.

## 2. Materials and Methods

We performed a retrospective cohort study of patients who underwent RN due to the presence of a kidney mass suspected of malignancy. The following data were considered: age, gender, body mass index (BMI), TNM staging, Fuhrman grading, hypertension, diabetes, and medical therapy (ACE inhibitors (ACEi), angiotensin II receptor blockers (ARBs), calcium antagonists, beta blockers, and diuretics). Serum creatinine (s-Cr) values (Kinetic Picrate standardized (COBAS C 800) for IDMS) were collected before surgery (t0), at 24 and 48 h after surgery, and at dismissal (respectively t1, t2, and tf), as well as after one year (ty) to detect renal function fluctuations and the subsequent risk of AKI, which usually presents within 48 h of surgery [9,10]. Based on the literature, a one-year follow-up was decided on, since this time window is usually the most accurate when establishing “stable” chronic renal function in a patient undergoing RN [8]. The glomerular filtration rate (GFR) was estimated at each time point using the creatinine-based estimated glomerular filtration rate (eGFR) formula: CKD-EPI [11]. We evaluated eGFR variation from the pre-surgical value to t1, t2, tf, and ty. eGFR categories were created according to the KDIGO [12] guidelines for G categories in GFR setting different thresholds: 90 mL/min/1.73 m^2^ (G1), 60 mL/min/1.73 m^2^ (G2), and 45 mL/min/1.73 m^2^ (G3a and G3b-4). The study consists in two different but related parts. In the first one, the primary outcome is to identify clinical variables correlated with the AKI onset after radical nephrectomy; in the second one, the primary outcome is to determine the eGFR decay after one year from surgery. In both cases subsequent analysis was carried out to model the eGFR decay (during hospitalization and after one year) with respect to the clinical variables.

The cohort consisted of 144 consecutive patients diagnosed with a kidney mass who were candidates for elective radical nephrectomy (2009–2019). Exclusion criteria were as follows: (a) age < 18 years, (b) CKD category G5 according to KDIGO guidelines [12], (c) solitary kidney, and (d) the diagnosis of medical nephropathy with the pathological analysis. The study was approved by the San Raffaele Hospital ethical committee as ‘PROTOCOLLO_RENE’ and all the participants gave their written informed consent.

Postoperative AKI was diagnosed according to s-Cr increase 72 h after surgery based on the KDIGO criteria [13]. According to this classification, a s-Cr increase higher than 1.5 times the baseline value was classified as stage 1, higher than 2 times as stage 2, and higher than 3 times as stage 3. Urinary output data were not collected for AKI evaluation. eGFR was not considered to determine AKI onset because the formulas are validated over stable values referring to chronic diseases or steady-state conditions [14,15,16].

Histological evaluation was performed on two dedicated specimens of kidney tissue derived from the unaffected part of the removed kidney (at least more than 3 cm away from the tumor). All histological samples were examined by the attending pathologist in order to identify signs of medical nephropathy. A slide with a specimen of approximately 15 × 20 mm containing both the medulla and cortex from normal tissue was prepared from histology. Then, two parallel lines were drawn on the normal kidney tissue with a marking pen to simulate biopsies using a 16-gauge biopsy core. A Nikon Eclipse 80i microscope (Nikon, Tokyo, Japan) with Pan Fluor was used at different power to evaluate an area of about 20 × 1.3 mm that was randomly chosen from one side of the line.

All samples were buffered, formalin fixed, paraffin embedded, and 4 mm thick kidney sections were stained with hematoxylin and eosin. We then analyzed samples for each subject using the chronic lesions score described by Remuzzi et al. in 1999 [17]. This score was originally designed to evaluate expanded donors in kidney transplantation and specifically looks for chronic damage. This chronicity score (CS) analyzes four microscopic anatomical areas—glomerular, tubular, interstitial, and arterial—and classifies four parameters as follows: (a) global glomerular sclerosis, (b) tubular atrophy, (c) interstitial fibrosis, and (d) arterial narrowing. All parameters are scored as 0 (absence), 1 (from 0% to 20%), 2 (20% to 50%), or 3 (>50%). A total score between 0 and 3 allows for a single kidney transplant, a total score between 4 and 6 suggests a double kidney transplantation, whereas a score > 7 contraindicates transplantation. The highest score between the two samples was considered for statistical analysis. The grouping variables used in our analysis included the presence of damage on the four parameters, taken individually, and the whole CS. Finally, for each patient we collected the highest score between the two samples. The patients were then divided into four groups according to their scores: Score 0, Score 1, Score 2, and Score ≥ 3.

Analyses were carried out to detect potential correlations between the outcomes of interest (AKI onset, eGFR decay) and other independent variables. A linear regression model was used to predict continuous variables (eGFR variation in hospitalization and follow-up), while we adopted the non-parametric Wilcoxon test (two groups) and Kruskal–Wallis test (more than two groups) for comparisons of continuous variables between groups. Wilcoxon test with Holm’s correction was used for post hoc test comparisons and Fisher’s exact test was performed for comparisons between categorical variables. Binomial logistic regression was used to identify possible predictors of AKI onset. The significance level for all the analyses was set at 0.05. All analyses were performed using programming language for statistical computing R version 3.6.3 (R Core Team, Vienna, Austria) [18], R package “Tidyverse” [19] and the free and open-source integrated development environment RStudio Version 1.2.5033 (RStudio Team, Boston, MA, USA) [20].

## 3. Results

### 3.1. AKI and eGFR Variation during Hospitalization

Descriptive characteristics of the patient population are reported in Table 1.

Overall, 21% of patients had CS = 0, 35% had CS = 1, 26% had CS = 2, and 18% had CS ≥ 3. No correspondence was observed with the TNM score (*p* = 0.7) or Fuhrman grade (*p* = 0.3). The median hospital stay after the surgery was 3 days (3–3). After surgery, 69% of patients demonstrated a rise in serum creatinine, which classified them as affected by AKI, according to KDIGO criteria. It is important to underline that the majority of these patients were classified as stage 1 (58%), with only a small percentage of stage 2 (8%) and 3 (3%). For the purposes of our analysis, we considered AKI onset as the outcome and investigated for predictors among clinical data and comorbidities. We did not observe any correlation with age, gender, intraoperative blood loss, presence of hypertension (considering the use of specific drugs), diabetes, BMI, TNM staging, Fuhrman grading, and histopathology. Logistic regression predicting AKI onset showed that baseline eGFR was a significant predictor for the CKD-EPI formula, as shown in Table 2.

**Figure 1:**
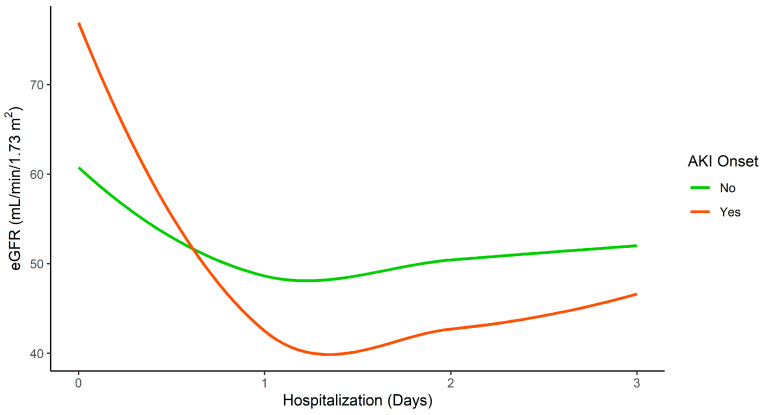
eGFR trend during hospitalization using Loess regression according to AKI onset.

We observed that the higher the baseline eGFR value, the greater the risk of AKI development during the first 72 h following RN (odds ratio (OR) 1.04 for CKD-EPI). Evaluation of the eGFR trend after nephrectomy showed that patients who developed AKI had a higher pre-surgery eGFR, as shown in Figure 1. 

Linear regression analysis between the pre-surgery value of eGFR and its decay at 24 h, 48 h, and 72 h exhibited significant results (Table 3). 

**Figure 2:**
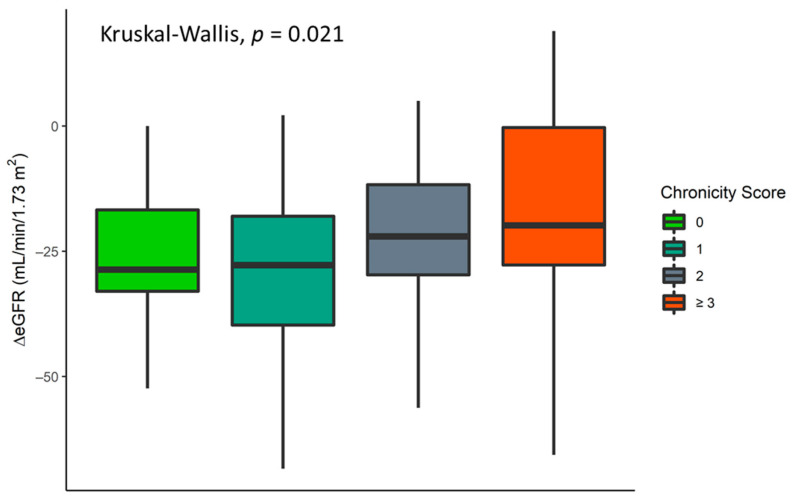
eGFR decay (baseline–discharge) distribution according to chronicity score (CS).

Considering the histology of the healthy part of the removed kidney, neither the CS nor its components taken individually proved to be good predictors of AKI on logistic regression. In the Kruskal–Wallis analysis, the eGFR decay, from baseline to discharge, correlated with CS (*p* = 0.02) and with the presence of tubular atrophy (*p* = 0.006) or interstitial fibrosis (*p* = 0.02). The results confirmed that already damaged tissue correlates with a milder eGFR decay (Table S1, Figure 2, Figure S1 in Supplementary Materials). Post hoc analysis, adopting Holm’s correction, revealed no differences among the scores.

### 3.2. eGFR Decay 1 Year after Radical Nephrectomy

**Figure 3:**
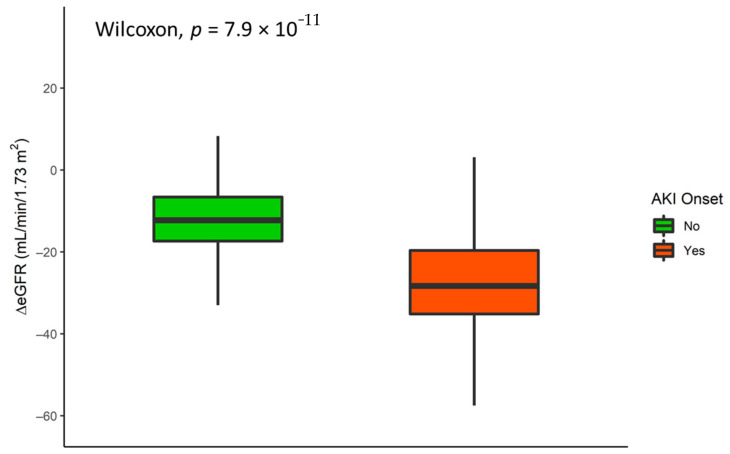
eGFR decay (baseline–after one year) distribution according to AKI onset.

One year after surgery, the median s-Cr was 1.36 mg/dL (1.12–1.67), and 2% of patients were classified (according to the CKD-EPI equation) as G1, 26% as G2, 58% as G3a, and 14% as G3b-4. No significant correlations regarding hypertension, diabetes, and overweight or obesity at time zero were observed. A significant correlation (*p* < 0.001) was found between baseline eGFR and renal function decay over 1 year. The higher the baseline value, the greater the eGFR decline. (Table 3). AKI onset demonstrated a significant correlation with eGFR decay (*p* < 0.001). Indeed, patients who developed AKI during hospitalization showed a greater than 17% decline (Figure 3).

**Figure 4:**
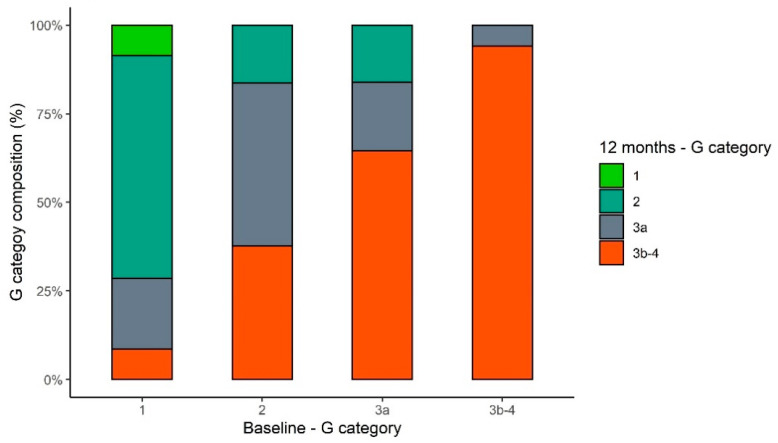
G category distribution after one year according to the baseline category.

We also observed that the eGFR decay after 1 year correlated with the CS of the removed kidney (*p* = 0.003). The post hoc test enlightened that eGFR decay for patients with CS ≥ 3 had a milder reduction of the renal function than patients with CS = 0 and 1. The four components of the CS were correlated with eGFR decline, as reported in Figure S2. AKI onset, hypertension, and the presence of arterial narrowing produced the most accurate model for multivariable analysis, as reported in Table 3. After one year from surgery, the change distribution to those belonging to the G category from CKD classification with respect to the starting category is reported in Figure 4.

## 4. Discussion

In the last decade the concepts of surgical post-operative AKI and CKD caused by the acute loss of nephrons have been proposed in order to distinguish them from the pathological medical conditions of AKI and CKD [8]. In fact, surgical patients displayed a completely different pattern of eGFR decline over time with respect to their nephrological counterparts [8]. We decided to study whether some clinical, pathological, and laboratory features might be related to the onset of AKI during hospitalization and to the decline in renal function at 12 months. eGFR was considered to be an indicator to evaluate kidney status, even though it should be used to diagnose chronic kidney disease and not acute damage [14,15,16], and it proved to be the best indicator in all the analyses and showed the highest number of significant correlations. We found a strong correlation between higher pre-operative eGFR and the augmented incidence of AKI. Patients with eGFR ≥ 60 mL/min/1.73 m^2^ displayed a median eGFR decay of 29 mL/min/1.73 m^2^ (20–34) and a 74% rate of AKI development. In parallel, another significant association was reported between lower CS, meaning the absence of histological damage, and a higher eGFR decline. These results are hypothesis generating. First, the absence of a strong correlation between pre-existing hypertension and AKI development could be related to good compliance with anti-hypertensive therapy by patients before surgery. Regarding obesity, a possible explanation could be that this condition likely leads to a compensation mechanism given its known effect on glomerular hyperfiltration. As far as diabetes is concerned, none of the patients had diabetic nephropathy with clear signs of kidney dysfunction and gross proteinuria or progressive kidney injury. Furthermore, the absence of correlation between AKI development and the aggressiveness of the tumor may be linked to the inability of cancer tissue to affect overall renal function, while the remaining “healthy” parenchyma tends to compensate. Conversely, the surprising association between a “healthier” kidney status before surgery and the greater risk of developing surgical AKI could be unexpected at first glance. However, as the literature has already demonstrated, this result could be explained by a compensatory mechanism already present in patients who had a previously compromised kidney status with an eGFR < 60 mL/min/1.73 m^2^. Hyperfiltration in the remaining kidney seems to occur progressively in the advanced stages of CKD and, thus, the acute loss of nephrons does not represent a severe traumatic event on overall renal function in comorbid patients. On the contrary, as demonstrated in the literature by the comparison between LKDs and oncological patients, surgical AKI is more likely to occur in healthy individuals with no histological damage because the total renal function is still carried out by both organs. The same observations were reported by Martini et al., who showed that partial nephrectomy induces more strain in patients with normal baseline eGFR [21]. Our results confirm these findings, although some aspects remain unclear. This evidence leads us to question whether the increase in serum creatinine represents a pathological condition named “surgical AKI” or if it is merely a natural consequence of the operation. The rise in creatinine could be related to either the loss of the functioning nephron units in the cancer kidney or to the insufficient compensatory mechanism carried out by the contralateral kidney. In this regard, removing the kidney could result in a physiological period of adaption during which the patient’s eGFR may appear to decrease but will eventually stabilize after one year [8]. Considering eGFR over time, our results demonstrated that the onset of AKI was correlated with a persistent eGFR reduction (*p* < 0.001). We observed that at 12 months, 85% of AKI patients fell into a lower eGFR category with respect to their baseline, while only 15% remained within the same category. Interestingly, the median eGFR decay in patients who developed AKI was greater among those whose eGFR was ≥60 mL/min/1.73 m^2^. As a matter of fact, the greater the impairment of baseline renal function, the lower the median decline in eGFR over time. In addition, none of the patients with baseline eGFR < 60 mL/min/1.73 m^2^ developed ESRD at 1 year. Conversely, patients with normal renal function had a median decay of 28 mL/min/1.73 m^2^ (21–34) one year after RN. Finally, it is also important to underline that the eGFR value at discharge after RN did not correlate with the decay at one year. The chronicity score evaluated the healthy part of the removed kidney at the time of surgery and demonstrated that the absence of histological damage (CS0–CS1) was prognostically related to a greater decline at 1 year, with worsening of the eGFR category in 87% of cases. Regarding the absence of arterial narrowing, 88% of patients developed CKD, while for kidney fibrosis, this finding occurred in 81% of patients. The correlation between AKI development and eGFR decay at 12 months confirmed the hypothesis that in some cases the remnant contralateral kidney is likely unable to compensate for the removed one in the short- to mid-term, especially if eGFR was higher and no histological damage was present at baseline. These results suggest that, before surgery, both kidneys were correctly filtrating in these patients and that the remaining kidney was not able to achieve the same eGFR total value on its own, neither immediately nor at 1 year. On the contrary, when baseline eGFR was <60 mL/min/1.73 m^2^, most of the overall renal filtration was being performed by the contralateral kidney, and so, acute and chronic decline remained low because the contribution of the tumoral kidney was negligible. These results can be compared to those reported by Bachrach and colleagues, who quantified the contribution of the removed kidney to overall filtration ranging from 50% to less than 20% in a study involving 136 patients who underwent radical nephrectomy or nephroureterectomy and had a preoperative renal scan with calculated differential function. The compensation of the remaining kidney exceeded 12% of the expected value in the entire population and almost 15% in patients with eGFR < 60 mL/min [22]. These data, consistently with those reported in our cohort, cannot be completely explained on the basis of current knowledge. Immediately after nephrectomy, compensation mechanisms including an increase in renal blood flow have been observed [23]. We can speculate that in CKD patients, these compensation mechanisms are “per se” hyperactivated in order to face the reduction in the nephronic mass and can more easily compensate the acute parenchymal loss. These hypotheses need to be specifically evaluated in dedicated trials. Another crucial point that our work clearly demonstrated is the importance of CS analysis, which is something that is not always performed by pathologists who focus more on the tumoral part. In fact, arterial narrowing or interstitial fibrosis in the presence or absence of kidney damage could be an important, novel parameter to take into consideration in the clinical follow-up and functional outcome. Histological evaluation could give additional insights to the real status of the kidney in combination with other parameters [24,25]. However, the authors believe that these results could also be re-evaluated from a different perspective if correction of the eGFR value after RN were applied, thus changing the viewpoint that a solitary kidney should act as two in terms of eGFR. For instance, we could propose a physiological decline for each category of eGFR after RN, both in acute and in chronic decline (Table 4), which could mitigate the interpretation of current classifications of AKI and CKD stages related specifically to patients who undergo kidney surgery (conservative or radical) in order to distinguish them from patients affected by medical nephropathies. It would be fundamental to classify oncological kidney patients in a new, more appropriate nephrological system, which would allow them to receive the correct designation of surgical AKI and surgical CKD.

The main limitations of the study are due to its retrospective nature. The use of serum creatinine values for classifying AKI was due to the lack of other biomolecular marker levels [10,26,27,28]. Furthermore, data regarding urine output were not available and were not used for AKI evaluation. However, s-Cr values were measured daily for the first 48 h after surgery and at discharge, conferring solidity to the definition of AKI according to KDIGO criteria. The analysis did not take into consideration the type of surgery, although the majority of enrolled patients underwent the same type, and this could be considered less influential in radical nephrectomy as compared to partial. The use of eGFR instead of measured GFR could also introduce some level of bias [29]. Regarding the linear and logistic models we obtained, the low coefficient of determination R^2^ values (for both models) indicate low accuracy and are mainly due to two factors: the small number of patients involved in the creation of the model and the biological variability that plays a key role compared to external elements [30].

## 5. Conclusions

Predicting AKI and eGFR decay after RN should take into consideration a combination of clinical, laboratory, and pathological features. The pathological analysis should be routinely performed in the healthy part of the removed kidney and combined with the other clinical parameters to provide tailored multidisciplinary counseling. Finally, both surgically derived AKI and CKD should be considered and classified differently with respect to their medical alter egos.

## Figures and Tables

**Table 1:** Baseline characteristics.

	Overall	G1	G2	G3a	G3b-4
Patients	144	35	61	31	17
Age—years (median, IQR)	68, 62–75	62, 52–66.5	68, 63–75	74, 65.5–77	78, 75–85
Male/Female ratio	2.4	2.2	2.1	4.1	2.4
BMI (median, IQR)	26, 24–28	26, 24–28	26, 24–29	26, 24–28	25, 23–28
Serum Creatinine t0—mg/dL (median, IQR)	1.0, 0.8–1.2	0.8, 0.6–0.8	0.9, 0.8–1.1	1.3, 1.2–1.4	1.7, 1.4–2.0
eGFR CKD-EPI t0—mL/min/1.73 m^2^ (median, IQR)	73, 57–90	97, 93–101	78, 67–82	55, 51–58	37, 30–41
Hypertension	63%	46%	66%	71%	71%
ACEi	16%	12.5%	17.5%	14%	17%
Beta-Blockers	22%	6.25%	22.5%	27%	33%
Calcium Antagonists	16%	12.5%	10%	18%	33%
Diuretics	13%	6.25%	12.5%	14%	25%
ARBs	17%	/	20%	14%	33%
Diabetes type II	22%	20%	23%	19%	29%
Intraoperative Blood Loss (median, IQR)	300, 100–700	200, 100–675	325, 100–613	400, 125–925	500, 200–1700
T (pTNM)					
1 (%)	42%	46%	47%	26%	47%
2 (%)	8%	11%	8%	7%	/
3 (%)	47%	43%	43%	61%	47%
4 (%)	3%	/	2%	6%	6%
Histopathology					
Clear Cell (%)	73%	79%	73%	72%	64%
Papillary (%)	9%	9%	5	16%	7%
Cromophobe Cell (%)	8%	9%	8%	9%	6%
Oncocytoma (%)	10%	3%	14%	3%	23%
Fuhrman grade					
1 (%)	2%	/	3%	3%	/
2 (%)	48%	48%	52%	42%	41%
3 (%)	33%	43%	28%	39%	24%
4 (%)	7%	6%	3%	13%	12%
Benign (%)	10%	3%	14%	3%	23%
AKI (KDIGO)					
1 (%)	58%	71%	57%	52%	41%
2 (%)	8%	11%	11%	3%	/
3 (%)	3%	6%	3%	3%	/
Chronicity Score					
0 (%)	21%	34%	25%	6%	6%
1 (%)	35%	49%	36%	32%	6%
2 (%)	26%	9%	30%	36%	29%
≥3 (%)	18%	8%	9%	26%	59%

**Table 2:** Univariable logistic regression for AKI onset.

Variable	Univariate Logistic Regression *p* Value	Odds Ratio (95% Confidence Interval)
S-creatinine t0 (per decimal units)	<0.001	0.81 (0.71–0.89)
eGFR CKD-EPI t0	<0.001	1.04 (1.02–1.06)
Age	1	
BMI	0.5	
Intraoperative Blood Loss	0.8	
Hypertension	0.09	
Diabetes	0.7	
Fuhrman Grade	0.4	
TNM Stage	0.8	
Histopathology	0.4	

**Table 3:** Single variable and multivariable linear regression for eGFR decay at 24 h, 48 h, dismissal, and one year after surgery.

	**Univariable Regression *p* Value**	**β (95% Confidence Interval)**	**R^2^**
24 h Decay			
eGFR CKD-EPI t_0_	<0.001	−0.52 (−0.61, −0.43)	0.49
48 h Decay			
eGFR CKD-EPI t_0_	<0.001	−0.45 (−0.55, −0.35)	0.36
72 h Decay			
eGFR CKD-EPI t_0_	<0.001	−0.44 (−0.53, −0.34)	0.35
1-year Decay			
eGFR CKD-EPI t_0_	<0.001	−0.42 (−0.52, −0.33)	0.35
1-year Decay			
eGFR CKD-EPI t_f_	0.3		
	**Multivariable Linear Regression *p* Value**	**β (95% Confidence Interval)**	**R^2^**
1-year Decay CKD-EPI			
AKI Onset	<0.001	15 (11, 20)	0.28
Arterial Narrowing	0.002	−8 (−3, −12)	
Hypertension	0.1	−3 (−8, 1)	

**Table 4:** Median eGFR decay by CKD G category.

**Acute Decay**	**Median Decay (IQR) mL/min/1.73 m^2^**	**Median Decay (IQR) %**
G1	37 (27–43)	36 (26–45)
G2	29 (19–35)	37 (28–46)
G3a	17 (1–24)	33 (2–42.5)
G3b-4	7 (2–14)	26 (4–36)
**Chronic Decay**	**Median Decay (IQR) mL/min/1.73 m^2^**	**Median Decay (IQR) %**
G1	31 (26–43.5)	31 (41–26.5)
G2	27 (18–33)	36 (42–27)
G3a	14 (6–19.5)	24 (34.5–11.5)
G3b-4	8 (1–20)	24 (50–4)

## Data Availability

The data presented in this study are available on request from the corresponding author.

## Supplementary Materials

The following are available online at https://www.mdpi.com/article/10.3390/jcm10153322/s1, Table S1: Kruskal–Wallis and Wilcoxon test for CS score and its components vs. eGFR decay (baseline–discharge). Figure S1: eGFR decay (baseline–discharge, Y-axis) distribution according to the presence of histological damage stratified based on the four components (interstitial fibrosis, glomerular sclerosis, tubular atrophy, and arterial narrowing) of the chronicity score (CS). Figure S2: eGFR decay (baseline–after one year, Y-axis) distribution according to the presence of histological damage stratified on the basis of the four components of the chronicity score (CS) (interstitial fibrosis, glomerular sclerosis, tubular atrophy, and arterial narrowing).
