# Supplementary material for: Unexpected Outcomes of Renal Function after Radical Nephrectomy: Histology Relevance along with Clinical Aspects
Source: J Clin Med. 2021 Jul 28;10(15):3322. doi: 10.3390/jcm10153322 (PMC8347310; doi:10.3390/jcm10153322)

Supplementary material

Supplementary Table S1 – Kruskal-Wallis and Wilcoxon test for CS score and its components vs eGFR decay (baseline – discharge)

Supplementary Figure S1 – eGFR decay (baseline – discharge, y axis) distribution according to the presence of histological damage stratified based on chronicity score (CS) four components (interstitial fibrosis, glomerular sclerosis, tubular atrophy, arterial narrowing).

Supplementary Figure S2 – eGFR decay (baseline – after one year, y axis) distribution according to the presence of histological damage stratified on the basis of the four components of the chronicity score (CS) (interstitial fibrosis, glomerular sclerosis, tubular atrophy, arterial narrowing).

Supplementary Table S1 – Kruskal-Wallis and Wilcoxon test for CS score and its components vs eGFR decay (baseline – discharge)

CKD-EPI Decay	*p value*
Chronicity Score	0.02
Glomerular Sclerosis	0.7
Interstitial Fibrosis	0.02
Tubular Atrophy	0.006
Arterial Narrowing	0.1

Supplementary Figure S1 – eGFR decay (baseline – discharge, y axis) distribution according to the presence of histological damage stratified based on chronicity score (CS) four components (interstitial fibrosis, glomerular sclerosis, tubular atrophy, arterial narrowing).

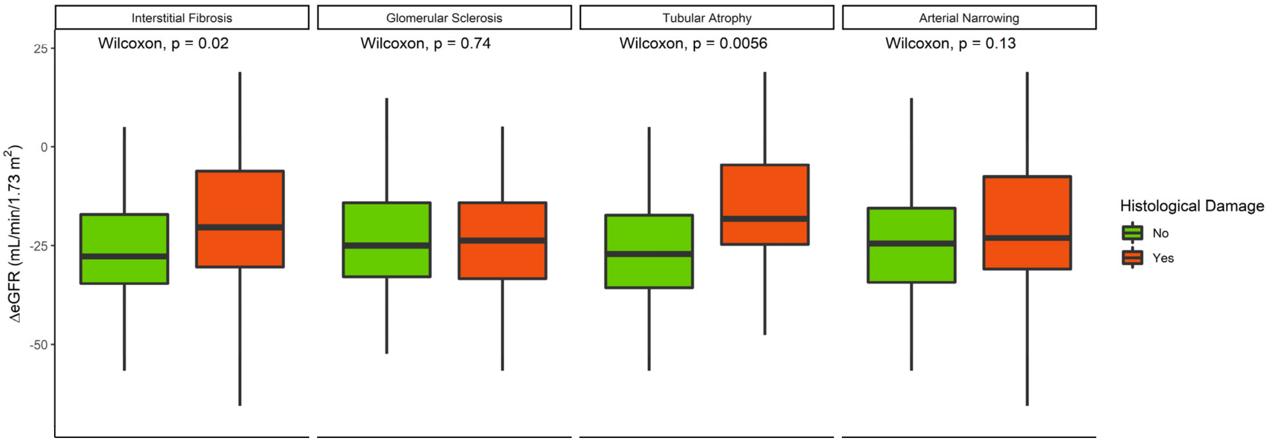

Supplementary Figure S2 – eGFR decay (baseline – after one year, y axis) distribution according to the presence of histological damage stratified on the basis of the four components of the chronicity score (CS) (interstitial fibrosis, glomerular sclerosis, tubular atrophy, arterial narrowing).

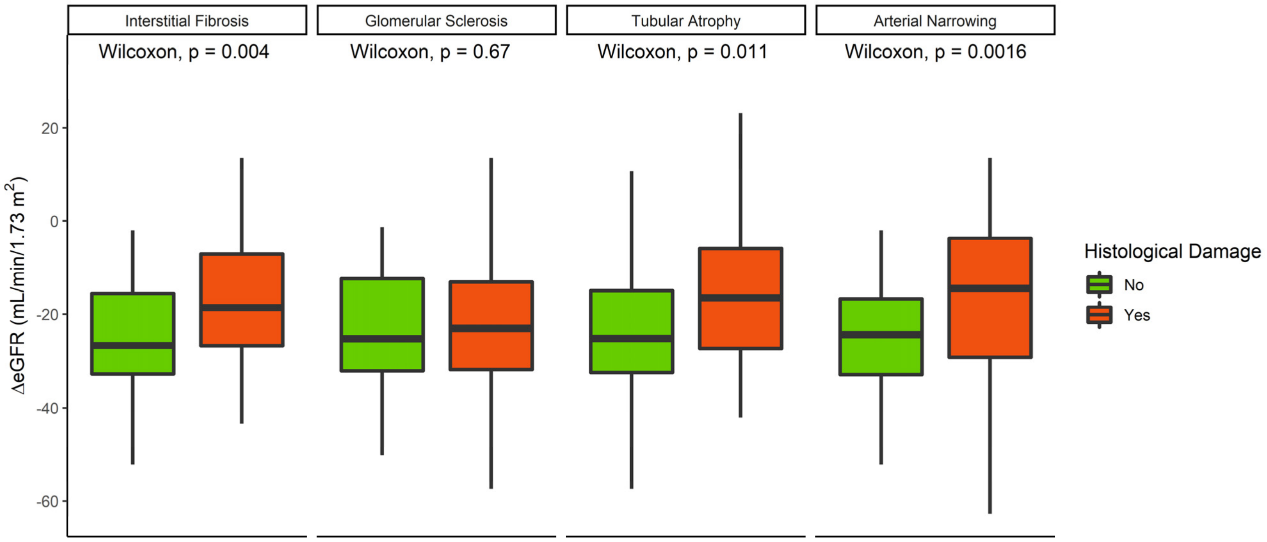

Supplement: Supplementary file 1 [file jcm-10-03322-s001.zip › jcm-1285426-supplementary.pdf]
